# Accessibility of Catering Service Venues and Adolescent Drinking in Beijing, China

**DOI:** 10.3390/ijerph120707208

**Published:** 2015-06-26

**Authors:** Shijun Lu, Songming Du, Zhoupeng Ren, Jing Zhao, Christina Chambers, Jinfeng Wang, Guansheng Ma

**Affiliations:** 1 National Institute for Nutrition and Health, Chinese Center for Disease Control and Prevention, 29 Nanwei Road, Xicheng District, Beijing 100050, China; E-Mails: lushijun666666@163.com (S.L.); dusm9709@126.com (S.D.); 2 State Key Laboratory of Resources and Environmental Information System, Institute of Geographic Science and Natural Resource Research, Chinese Academy of Sciences, A11 Datun Road, Chaoyang District, Beijing 100101, China; E-Mail: renzp@lreis.ac.cn; 3 Beijing Chaoyang District Center for Disease and Prevention, 25 Huaweili, Chaoyang District, Beijing 100021, China; E-Mail: cycdc2012@163.com; 4 Department of Pediatrics, Family Medicine and Public Health, Skaggs School of Pharmacy and Pharmaceutical Sciences, University of California San Diego, 9500 Gilman Drive, La Jolla CA 92093, USA; E-Mail: chchambers@ucsd.edu; 5 Department of Nutrition and Food Hygiene, School of Public Health, Peking University, 38 Xue Yuan Road, Haidian District, Beijing 100191, China

**Keywords:** adolescent drinking, catering service venues, alcoholic beverage, accessibility, school environment

## Abstract

This study assessed the association between accessibility of catering service venues and adolescents’ alcohol use over the previous 30 days. The data were collected from cross-sectional surveys conducted in 2014, 2223 students at 27 high schools in Chaoyang and Xicheng districts, Beijing using self-administered questionnaires to collect the adolescents information on socio-demographic characteristics and recent alcohol experiences. The accessibility of, and proximity to, catering service venues were summarized by weights, which were calculated by multiplication of the type-weight and the distance-weight. All sampled schools were categorized into three subgroups (low, middle, and high geographic density) based on the tertile of nearby catering service venues, and a multi-level logistic regression analysis was performed to explore variance between the school levels. Considering the setting characteristics, the catering service venues weighted value was found to account for 8.6% of the school level variance of adolescent alcohol use. The odds ratios (OR) and 95% confidence intervals (CI) of drinking over the past 30-days among adolescents with medium and high accessibility of catering service venues were 1.17 (0.86, 1.57) and 1.47 (1.06, 2.02), respectively (*p* < 0.001 for trend test). This study addressed a gap in the adolescent drinking influence by the catering service venues around schools in China. Results suggest that the greater accessibility of catering service venues around schools is associated with a growing risk of recent drinking.

## 1. Introduction

Adolescent drinking is considered one of the most serious public health concerns, and more than sixty percent of high school students have reported consuming alcohol in the United States [1], Europe [2] and Asia [3,4], including China [5]. To our knowledge, underage drinking is associated with a wide array of negative outcomes, such as adolescent violence, suicide, drug use and other alcohol-related consequences [6,7,8], and may exert a cascade effect on adulthood health and development [9,10,11].

There is an increasing volume of research in the literature identifying many risk factors that are associated with adolescents’ alcohol use. These risk factors include temperament traits, parental and peer influences, social background, genetic factors, as well as media advertisements [12,13,14]. It is worth noting that the geographic density of alcohol outlets is also considered to be an important factor related to alcohol consumption [15]. Thus, restricting adolescents’ access to alcohol is a commonly advocated approach to control underage drinking and related adverse outcomes [16].

Few studies have assessed associations between physical accessibility, such as alcohol outlet density, and individual level of adolescents’ alcohol use. In one ecological multilevel analysis, a positive association was found between the number of outlets within two miles of a school campus and student binge drinking rates [17]. In a Swiss study examining this relationship, alcohol outlet density was also found to be associated with adolescent drinking behavior [18]. Similar findings have been reported in surveys on campus surroundings for college students [19], and the rationale of this strategy is that reducing adolescents ability to obtain alcoholic beverages will reduce consumption [20].

In China, students are not usually forced to have meals at school, and they are allowed to choose catering service venues, such as restaurants, porterhouses, and snack bars, around the schools by themselves. This close proximity, combined with loose enforcement practices for verifying the age qualifications of alcohol purchasers, suggests that catering service venues may represent a major source of alcoholic beverages for adolescents. Therefore, a large number of catering service venues that provide daily meals for students appear in areas surrounding schools, and these have made alcohol more accessible to students. To our knowledge, there have been no studies published regarding the physical accessibility of catering service venues surrounding school environments in relation to adolescents’ drinking behavior in China. We hypothesized that the number and proximity of catering service venues surrounding schools in China would influence students’ daily alcohol use.

## 2. Methods

### 2.1. Participants and Procedure

**Figure 1:**
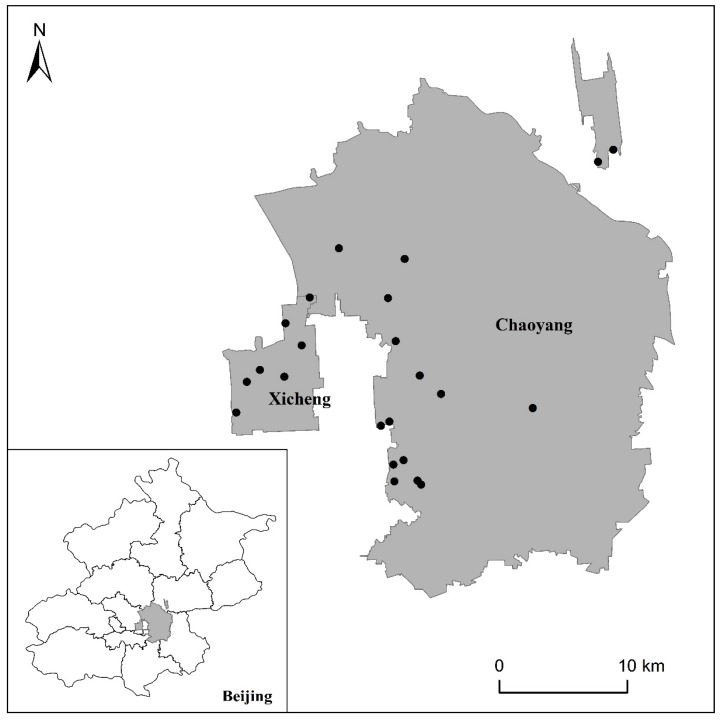
The black points show the sampling schools located in Xicheng and Chaoyang Districts, Beijing.

Individual student level data for this analysis was collected through questionnaires completed in May 2013. The survey sample included 2449 full-time students from 27 high schools in Chaoyang and Xicheng districts in Beijing, which are illustrated in Figure 1. Two classes from the first and second grades within each school (G7, G8 for junior high school and G10, G11 for regular and vocational senior high school) were selected for the study. The study protocol was approved by the Ethics Committee for Research in Human Subjects of the National Institute for Nutrition and Health, Chinese Center for Disease Control and Prevention (2013-019), and written informed consent was given before the survey, and obtained from all participating students and their guardians.

### 2.2. Individual Level Variables

#### 2.2.1. Outcome Variables

The outcome for this study was recent alcohol use, which was defined as any reported drinking during the past 30 days prior to the survey, regardless of the quantity of alcohol consumed. The outcome variable was indicated by a dummy variable (yes/no).

#### 2.2.2. Demographic Characteristic Variables

Based on the literature, variables that could be potential confounders were selected from the survey. These included gender, school type, school performance, smoking, family structure, and socioeconomic status (SES).

Sex was indicated by a dummy variable (male/female). For the school type variable, regular and vocational senior high schools were combined into one category because the students’ age range was similar, with junior high school as a separate category. School performance of the respondents was based on grades on the most recent semester final exam, and was divided into three levels using exam score tertiles as the cut-point. Tobacco use at anytime was indicated by a dummy variable (yes/no).

Family structure was classified in three categories: adolescent living with both biological parents; adolescent living with only the mother or the father; or adolescent living with other persons, excluding their biological parents.

The family affluence scale (FAS) was used as an alternative measurement of student’s family SES. This scale was chosen because annual household income is not available in China. FAS has been found to be reliable in that students can report accurately on the component items in agreement with the reports of their parents [21,22,23]. The FAS scale is based on four indicators: does your family own a car? (0, 1, 2, or more); how many times did you travel away on holiday with your family during the past 12 months? (0, 1, 2, 3 or more); do you have your own bedroom for yourself? (0, 1); and how many computers does your family own? (0, 1, 2, 3, or more). The final FAS score was calculated by adding the responses to these four indicators, which ranged from 0 to 9. The FAS scores were subsequently recoded into tertiles within each district (high, middle, low).

### 2.3. School-Level Variables

The accessibility of near-premises alcohol outlets was assessed using a 1 km Euclidean buffer (straight-line, circular of 1 km radius) around each sampled school, which is considered an empirically determined measure of easy walking distance of approximately 12~15 min at 4~5 km per hour [24,25,26]. A total of 2603 catering service venues was obtained using the geographic information system (GIS) layer of Xicheng and Chaoyang relative to outlet locations, inclusive of restaurant, hotel, eatery, snack bar, and dining hall venues.

**Figure 2:**
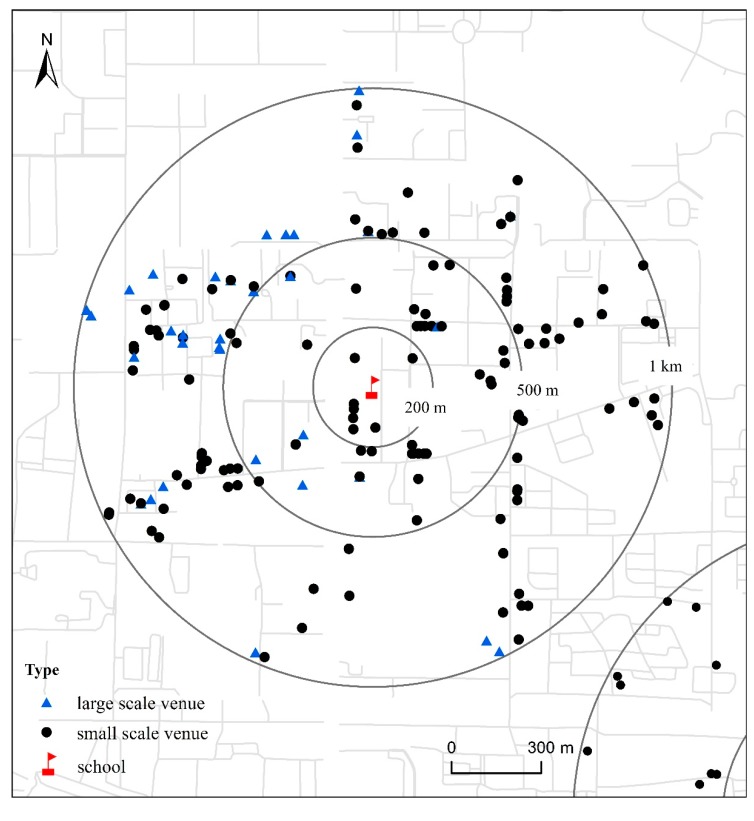
The distribution of catering service venues and different distance buffer for one school.

### 2.4. Measures

The point locations of the 27 sampled schools were geocoded based on their official addresses by using Google Earth. We then used ArcGIS version 10.2 to create 200-m, 500-m and 1-km buffers around each geocoded school point. After the buffers were created, we employed a spatial join to count the number of catering service venues within each of these 200-m, 500-m and 1-km buffers (Figure 2). The catering service venues were given different type-weights and distance-weights, according to the type of venue level and distance from each school. Experts are discussed based on the scale and the cost per person. A type-weight of 1 was assigned for those small scale venues that offered food or drink at a relatively lower cost, such as an eatery or snack bar, and a type-weight of 0.6 was assigned to the large scale venues that were considered to be more expensive outlets such as a hotel. A distance-weight of 1 was assigned for those venues with distances smaller than 200 m from the school, 0.75 for those between 200 m and 500 m and 0.5 for those between 500 m and 1 km [27]. The final weight of each catering service venue was calculated by multiplication of the type-weight and the distance-weight. The final weighted value for accessibility of catering service venues for each school was calculated by the summation of the final weights for venues associated with that school. The following formula could be used to calculate the different weights.


Weight _final_ = Weight _type_ × Weight _distance_ (1)

Weight _school_ = Weight _final 1_ + Weight _final 2_ + ...... + Weight _final n_ (2)

The value of Weight _type_ are 0.6 or 1. The value of Weight _distance_ are 0.5, 0.75 or 1. The final weights across all 27 schools (Weight _schools_) were subgrouped accessibility into three levels using the following tertiles as the cut-points: low (<41.8), medium (41.8~60.0) and high (>60) accessibility. The means of the weighted number of catering service places for the low, medium and high levels of accessibility were 28.1, 52.6 and 71.7, respectively.

### 2.5. Statistical Analysis

Data were missing on a number of key variables for some students including school performance score (n = 35), smoking (n = 87), SES as measured by FAS (n = 104), and these subjects were excluded from the analyses. We used logistic regression modeling to explore possible confounding due to the association of the demographic characteristics with recent alcohol use in adolescents. Due to the multi-stage sampling procedure, all odds ratios (OR) and 95% confidence intervals (95% CIs) were weighted by the probability of selection. SAS version 9.4 statistical software was used for this part of the analyses.

Next, a two-level random effects logistic regression was employed to analyze the hierarchically structured data. The first-level was the individual (n = 2223), and the second-level was the school (n = 27). This method allowed us to explain the second-level variations and to estimate correctly the variance for the outlet density parameter. At first, we constructed a null model to examine whether or not the prevalence of recent drinking of adolescents varied from school to school. Second, the five potentially confounding variables were added to the model to assess their effects on the outcome variable (recent alcohol use). Finally, we added the second-level explanatory variable to the model.

To evaluate the clustering of alcohol use within schools and the effect of the second-level explanatory variable, we applied several indicators of variation or clustering. At first, the median odds ratio (MOR) was calculated. The MOR quantifies the school-level variance into a form that is similar to the OR by comparing two students from each of two randomly selected schools, and the MOR is the median of the ORs for all possible pairs of students. The MOR is always greater than one, and if it is equal to one, there is no variation of adolescent alcohol use between schools. It can be computed with the following formula:

(3) $$\text{MOR}=\text{exp}\left[\sqrt{2\times V_{A}}\times 0.6745\right]\approx \text{exp}\left(0.95\sqrt{V_{A}}\right)$$

where *V_A_* is the school level variance, and 0.6745 is the 75th percentile of the cumulative distribution function of the normal distribution (mean 0, variance 1). To evaluate the second-level variance between schools, we calculated the proportional change in variance (PCV) [28,29]. The PCV is calculated as:

(4) $$\text{PCV}=\left(V_{A}-V_{B}\right)/V_{A}\times 100$$

where *V_A_* has the same meaning with Equation (3), and *V_B_* is the variance with more terms in the model. We used the MLwiN software (version 2.30) for the multilevel analysis of the school background characteristics in relation to recent alcohol use.

## 3. Results

Among the 2223 students available for the analysis, 533 reported alcohol consumption in the past 30 days (weighted prevalence: 22.1%). Table 1 displays the association of the selected individual variables in relation to recent alcohol use. The following variables were associated with past 30 days alcohol use: gender, regular/vocational senior high school, school performance, smoking, family structure, and SES.

**Table 1:** Univariate association of demographic characteristics with past 30 days alcohol use among 2223 adolescents in Beijing.

Variable	Non-Use (n = 1690) n (%_wt_)	Past 30 Days Alcohol Use (n = 533) n (%_wt_)	OR (95% CI)
Male gender (*versus* female)	760 (44.6)	288 (53.6)	1.24 (0.96,1.60)
High/vocational school (*versus* middle school)	773 (39.6)	346 (56.4)	1.70 (1.26,2.28) *
School examination score			
Medium (*versus* low)	837 (42.7)	273 (43.6)	1.32 (1.00,1.74)
High (*versus* low)	346 (18.1)	165 (27.8)	1.68 (1.32,2.14) *
Smoking	288 (13.5)	260 (38.3)	3.69 (2.75,4.96) *
Living with			
Single-parent (*versus* both parents)	246 (14.2)	92 (15.9)	1.04 (0.72,1.51)
Others (*versus* both parents)	115 (6.38)	65 (11.0)	1.54 (1.04,2.29) *
SES			
Middle (*versus* low)	860 (50.2)	266 (48.9)	1.18 (0.84,1.65)
High (*versus* low)	537 (32.3)	183 (36.2)	1.46 (1.04,2.04) *

* *p* < 0.05.

The findings of the multi-level analyses are shown in Table 2. The MOR at the school level of the null model (1.70) indicates an effect of school on the prevalence of recent alcohol use. In the model that included individual variables only, the predictors and their estimated effect sizes were similar to those found in the weighted logistic regression analysis, with the exception of gender. Compared to the null model, the school-level MOR in the model with individual variables decreased from 1.70 to 1.34, and the corresponding school-level PCV was 69.1%.

When the setting variable, the accessibility of catering service venues, was then added to the model, the reduction in the school-level variance decreased further to 77.7%. In particular, the OR for recent alcohol use among those with the high level of accessibility of catering service outlets was significantly higher than one. The trend test indicated that there was a significantly increased risk for recent alcohol use as the accessibility of catering service venues increased.

To evaluate further the influence of compositional effects when studying the contextual factor, we ran a model that included only the weighted variable for catering service venues’ accessibility. This model resulted in ORs and 95% CIs of 1.08 (0.67–1.74) and 2.14 (1.34–3.44) for recent alcohol use in students of schools with medium and high accessibilities, respectively. These estimates were not very different from those shown in Table 2 when compositional variables were added into the model, *i.e.*, 1.00 (0.69, 1.44) and 1.52 (1.03, 2.22) for schools with medium and high accessibilities, respectively.

**Table 2:** Predictors of past 30 days alcohol use among 2223 adolescents in Beijing.

Variables	Null Model OR (95% CI)	Model with Individual Demographic Variables only aOR (95% CI) ^a^	Model with Individual and School Setting Variables aOR (95% CI) ^a^
Male gender (*versus* female)		1.27 (1.02,1.57) *	1.29 (1.04,1.60) *
High/vocational school (*versus* middle school)		1.79 (1.31,2.45) *	1.56 (1.14,1.24) *
School examination score			
Medium (*versus* low)		1.18 (0.93,1.50)	1.18 (0.93,1.50)
High (*versus* low)		1.55 (1.17,2.06) *	1.55 (1.17,2.06) *
Smoking		3.42 (2.71,4.32) *	3.41 (2.70,4.30) *
Living with			
Single-parent (*versus* both parents)		1.08 (0.81,1.44)	1.07 (0.81,1.43)
Others (*versus* both parents)		1.53 (1.07,2.19) *	1.52 (1.07,2.17) *
SES			
Medium (*versus* low)		1.15 (0.85,1.56)	1.17 (0.86,1.57)
High (*versus* low)		1.43 (1.04,1.97) *	1.47 (1.06,2.02) *
**Setting variable**			
Accessibility of catering service venues			
Medium (*versus* low)			1.00 (0.69,1.44)
High (*versus* low)			1.52 (1.03,2.22) *
Trend test			*p* < 0.001
**Measures of variation or clustering**			
School level variance (SD)	0.314 (0.103)	0.097 (0.047)	0.070 (0.040)
PCV		69.1%	77.7%
MOR	1.70	1.34	1.29

* *p* < 0.05; **^a^** 95% CI for each of the covariates adjusted for all other variables.

## 4. Discussion and Conclusions

This study examined the association between accessibility of catering service venues around school settings with recent alcohol use reported by adolescents attending those schools. Findings from the multilevel analyses indicated that after controlling for individual-level factors, the high density of catering service venues surrounding schools was significantly and positively related to adolescents’ drinking in the past-30 days. These findings can provide useful information for policy makers about the alcohol environment as it relates to prevention of adolescent alcohol use.

Our findings suggest that the catering service venues around schools maybe one source of alcohol for adolescents. Some reasons for this may be the following. Due to the limited rest time provided in the students’ daily schedule, the areas immediately surrounding their schools are the main locations for students’ social activities, such as having meals and spending regular time together. Importantly, nearly all the catering service venues identified near the sampled schools offered alcohol for sale to their customers. Although signage in these venues is required to indicate that “selling alcohol to minors is prohibited”, there is no strict enforcement and no supervision of staff at such outlets to prevent them from selling alcoholic beverages to juveniles.

There are an increasing number of studies that utilize the GIS approach to investigate the spatial associations between substance use and ecological factors [30,31,32]. In our study, we calculated the density of accessibility catering service venues located around schools and assessed its relationship to the students’ recent alcohol use. The distance weights of catering service venues relied on Euclidean buffers rather than network buffers. Because we only aimed to explore access to catering service venues, we used a school buffer, which represented the distance that a pedestrian could walk within 15 minutes. However, there are many short cuts or unofficial footpaths that exist around schools. Thus, it would have been difficult to calculate an accurate network buffer.

There are several limitations to the present study. First, we only evaluated the relationship between the catering service venues around the school and did not consider the possible contribution of the other types of outlets, such as bars, pubs, and convenience stores. Second, some students who lived in close proximity to their school may be have obtained their meals at or closer to home; we did not consider the possible influence of the outlet density in the students’ residential neighborhood on recent alcohol use. Third, the values of the type weights of the catering service venues are recommended by the expert group in the Chinese society background. Some quantitative approaches to set the type weights values are needed. Finally, the sample selected may not have been representative of all adolescents in the whole city, especially for the suburban area.

These findings have implications for adolescent alcohol use prevention. Previous studies have reported that limiting on-premises alcohol outlet density is an effective strategy in lowering alcohol use, and is even preventative for several forms of injury and violence outcomes related to alcohol consumption [32,33]. Although, there are no laws for limiting the distribution of the drinking establishments around schools in China, many other approaches could be considered. First, eating venues surrounding schools, just like on-premises alcohol outlets, need to more stringently abide by laws that prohibit minors from purchasing alcoholic beverages, especially some severe punishments for the alcohol sellers around the schools. Second, the government alcohol licensing authority should pay more attention to the old alcohol sellers around schools or new applicants next to schools, including catering service venues, bars, pubs, and stores and supermarkets, and limited the total quantity of alcohol sellers.

The study showed that the greater accessibility of catering service venues around schools is associated with a growing risk of recent drinking over the previous 30-days among adolescents. Future studies are needed to determine how alcohol outlet density around residential neighborhoods may impact consumption, and/or if the distribution of outlets between school and home is related to drinking trajectories among youngsters. In addition, surveys should assess the effectiveness of alcohol control policies and official enforcement measures aimed to reduce social accessibility of alcohol to adolescents.
